# Performance of the 2024 McDonald Criteria in Patients Under Evaluation for Suspected Multiple Sclerosis

**DOI:** 10.1212/WNL.0000000000214688

**Published:** 2026-02-13

**Authors:** Wallace J. Brownlee, Davide Maccarrone, Riccardo Nistri, Charmaine Yam, Heather Wilson, Sarah Wright, Kshitij Mankad, Cheryl Hemingway, Alan J. Thompson, Yael Hacohen, Ahmed T. Toosy, Olga Ciccarelli

**Affiliations:** 1 Queen Square Multiple Sclerosis Centre, Department of Neuroinflammation, UCL Institute of Neurology, London, United Kingdom;; 2 NIHR University College London Hospitals Biomedical Research Centre, United Kingdom;; 3 Department of Human Neurosciences, Sapienza University of Rome, Italy;; 4 Great Ormond Street Hospital for Children, London, United Kingdom; and; 5 Dunedin Hospital Te Whatu Ora Southern, New Zealand.

## Abstract

**Background and Objectives:**

The 2024 McDonald criteria provide new recommendations for multiple sclerosis (MS) diagnosis, but validation of the criteria is lacking. We wanted to investigate the application and performance of the 2024 McDonald criteria in patients with suspected MS seen in routine clinical practice.

**Methods:**

We retrospectively identified patients referred with clinical and/or radiologic suspicion for MS between January and December 2024. All patients had a minimum diagnostic evaluation with brain and spinal cord MRI. The 2017 McDonald criteria were applied prospectively, and the 2024 McDonald criteria were applied retrospectively after the initial diagnostic workup and at last follow-up. We investigated (1) the number of patients diagnosed with MS using the 2017 McDonald criteria or the 2024 McDonald criteria, and (2) the diagnostic performance of the 2024 McDonald criteria when applied during the initial diagnostic workup, with an MS diagnosis using the 2017 McDonald criteria as the reference-standard.

**Results:**

In 347 patients (mean age 39.4 years, 66% female) with suspected MS followed up for mean 15.3 (range 8–20) months, 73 (21%) had alternative disorders and were excluded. After applying the 2024 McDonald criteria in the remaining 274 patients, more patients were diagnosed with MS after the initial diagnostic workup (220 vs 172, *p* < 0.01), and at follow-up (237 vs 204, *p* < 0.01), compared with the 2017 McDonald criteria, and fewer patients were diagnosed with clinically or radiologically isolated syndrome. The median time to diagnosis was 40 vs 84 days (*p* < 0.01), respectively. After initial diagnostic workup, the 2024 McDonald criteria demonstrated high sensitivity (92.6%, 95% CI 88.1%–95.8%) and accuracy (83.6%, 95% CI 78.7%–87.8%), for an MS diagnosis using 2017 McDonald criteria at last follow-up, but moderate specificity (57.8%, 95% CI 45.4%–69.4%). The increased rate of MS diagnosis and the diagnostic performance of the revised criteria was similar in children (<18 years) and older adults (>50 years).

**Discussion:**

Use of the 2024 McDonald allows for earlier MS diagnosis, but also more frequent diagnosis including in patients with radiologically isolated syndrome. The revised criteria have similar performance across the lifespan. Limitations include the lack of data on central vein sign and kappa free light chains, and missing optic nerve assessment in some patients.

## Introduction

Early diagnosis and treatment of patients with multiple sclerosis (MS) is essential to optimize long-term outcomes.^1^ Over the past 25 years, MS diagnostic criteria have been regularly updated incorporating new evidence on the use of MRI and other paraclinical tests.^2^ Although these changes have enabled earlier MS diagnosis,^3,4^ delayed diagnosis and misdiagnosis of MS, remain important challenges.^5^

The 2024 McDonald criteria provide updated recommendations for MS diagnosis.^6^ Key updates include (1) inclusion of optic nerve lesions detected by orbital MRI, visual evoked potentials (VEPs) and/or optical coherence tomography (OCT) as the fifth region in dissemination in space (DIS),^7,8^ (2) dissemination in time (DIT)/positive oligoclonal bands (OCBs) in CSF is not mandatory if patients have DIS in ≥4 regions,^9^ (3) new MRI biomarkers including central vein sign (CVS),^10-13^ and paramagnetic rim lesions (PRLs),^13-15^ (4) kappa free light chains (k-FLCs) as an alternative to CSF-specific OCBs,^16,17^ (5) removal of the need for a “typical” clinical syndrome to apply MS diagnostic criteria, including in patients with radiologically isolated syndrome (RIS)^18^ and nonspecific presentations,^19^ (6) a combined framework for MS diagnosis across the lifespan with special considerations in children, older adults, and patients with vascular comorbidities,^20^ and (7) a unified approach to MS diagnosis in patients with relapsing-remitting MS (RRMS) and primary progressive MS (PPMS).^21^

Elements of the revised McDonald criteria have only been tested in isolation,^7,9-12,14-16^ rather than in aggregate.^22^ Furthermore, the evidence that underpins the recent revisions largely comes from research cohorts that may not be representative of patients seen in routine clinical practice.^7,9-12,14,15,21^ We aimed to investigate the application and performance of the 2024 McDonald criteria in a contemporary cohort of patients under evaluation for suspected MS to understand their effect on MS diagnosis in clinical settings.

## Methods

### Patients

We retrospectively identified consecutive patients referred to MS specialist neurologists at the National Hospital for Neurology and Neurosurgery and Great Ormond Street Hospital with suspected MS, between January 1 and December 31, 2024. We included all patients with clinical and/or radiologic suspicion of MS and all patients had a minimum diagnostic workup with brain and spinal cord MRI. We excluded patients with an established diagnosis of MS, and in children, we also excluded patients with acute disseminated encephalomyelitis and/or positive myelin oligodendrocyte glycoprotein immunoglobulin G (MOG-IgG).

### Data Collection

Patient demographics, clinical presentation, and the results of paraclinical tests performed as part of routine clinical care were extracted from the electronic health record. Brain and spinal cord MRI were used to identify periventricular, cortical/juxtacortical, infratentorial, and spinal cord lesions, and new lesions on follow-up MRI scans, as reported by neuroradiologists. The results of other paraclinical tests were collected if available, including (1) postcontrast T1-weighted MRI looking for gadolinium-enhancing lesions, (2) orbital MRI to detect optic nerve involvement, (3) full-field pattern reversal VEPs, as reported by a neurophysiologist using local normative data, (4) OCT with an inter-eye difference in retinal nerve fiber layer of ≥6 μm and/or ganglion cell-inner plexiform layer of ≥4 μm considered abnormal, and (5) CSF analysis using isoelectric focusing combined with immunoblotting to detect CSF-specific OCBs. MRI techniques sensitive to the detection of CVS/PRLs and CSF analysis for k-FLC index were not routinely available at our hospitals at the time the patients were assessed.

### MS Diagnostic Criteria

Treating neurologists applied the 2017 McDonald criteria^23^ prospectively at the initial diagnostic evaluation and at follow-up, after excluding alternative disorders. The 2024 McDonald criteria^6^ were then applied retrospectively using clinical and paraclinical data after the initial diagnostic workup and at last follow-up. Specific recommendations for MS diagnosis in children, older adults, and patients with vascular comorbidities were considered.^6^

Patients were classified as having “possible MS” when there was suspicion of MS but the McDonald criteria were not satisfied and there was no better explanation, including patients with clinically isolated syndrome (CIS)^2^ and RIS (defined as ≥1 typical demyelinating lesion on MRI suggestive of MS^24^).

### Statistical Analysis

Data were analyzed using descriptive statistics with normally distributed continuous variables presented as means (SD), continuous variables as medians (ranges), and categorical variables as percentages. Diagnoses made in the same patient using the 2017 and 2024 criteria were compared using the McNemar test for paired proportions. To evaluate time to MS diagnosis, a shared frailty model^25^ (an extension of the Cox proportional hazards model designed for correlated data) was used to account for the paired nature of observations. The model assumed a gamma distribution for the frailty term and clustered by patient to handle the application of different diagnostic criteria in the same patient. Hazard ratios (HR) and 95% CIs were calculated to quantify the association between the diagnostic criteria and the rate of MS diagnosis. The significance of the frailty component was assessed with a likelihood-ratio test.

We estimated the performance of the McDonald 2024 criteria when applied at the initial diagnostic workup by calculating the sensitivity, specificity, accuracy, positive predictive value (PPV), and negative predictive value (NPV), with 95% CIs. We used 2 reference standards for performance testing (1) MS diagnosis using the 2017 McDonald criteria at follow-up and (2) new T2 lesions at follow-up.

We investigated the application and performance of the 2024 McDonald criteria first in the whole cohort and then separately in children (<18 years), older adults (≥50 years), and patients with vascular comorbidities (hypertension, smoking, diabetes, hyperlipidemia, or known macrovascular disease). We also analyzed the performance of the 2024 McDonald criteria in the whole cohort, including people referred with suspicion of MS but ultimately diagnosed with other disorders, although this is against the recommended approach to MS diagnosis where better explanation are excluded before applying MS diagnostic criteria.

Statistical analyses were performed using R. Statistical significance is reported at *p* < 0.05 (2-sided).

### Standard Protocol Approvals, Registrations, and Patient Consents

This study was approved as a service evaluation project by University College London Hospitals NHS Foundation Trust (17-202526-SE). Individual patient consent was waived for collection and analysis of data collected retrospectively as part of routine clinical care.

### Data Availability

The corresponding author has full access to all of the data in the study and takes responsibility for the data integrity and data analysis. The anonymized data set is available from the corresponding author on reasonable request.

## Results

### Patient Characteristics

We identified 347 patients referred with clinical and/or MRI suspicion of MS. The patient characteristics are summarized in Table 1. The mean (SD) age was 39.4 (13.9) years, two-thirds were female, and approximately one-third were from a Black, Asian, or other minority ethnic background.

**Table 1:** Patient Characteristics

	Patients with possible MS (n = 274)	Patients with alternative diagnoses (n = 73)
Age, y, mean (SD)	38.0 (13.8)	45.8 (12.6)
Female, n (%)	180 (66)	48 (66)
Non-White ethnicity, n (%)^a^	87/266 (33)	18/72 (25)
Vascular comorbidities, n (%)	48 (18)	28 (38)
Typical clinical syndrome, n (%)	223 (81)	9 (12)
Topographical involvement, n (%)		
Optic nerve	55 (20)	10 (14)
Spinal cord	122 (45)	4 (5)
Brainstem/cerebellar	50 (18)	6 (8)
Supratentorial	12 (4)	5 (7)
Other	35 (13)	48 (66)
Periventricular lesion(s), n (%)	237 (86)	21 (29)
Cortical/juxtacortical lesion(s), n (%)	153 (56)	5 (7)
Infratentorial lesion(s), n (%)	129 (47)	6 (8)
Spinal cord lesion(s), n (%)	198 (72)	13 (18)
Optic nerve lesion(s), n (%)^a,b^	77/166 (47)	8/32 (25)
Dissemination in space, n (%)		
0/5 regions	0 (0)	30 (41)
1/5 region	33 (12)	35 (48)
2/5 regions	65 (24)	7 (10)
3/5 regions	85 (31)	1 (1)
≥4/5 regions	91 (33)	0 (0)
Dissemination in time on MRI, n (%)^a,c^	53/250 (21)	2/48 (4)
CSF-specific oligoclonal bands, n (%)^a^	186/227 (82)	4/40 (10)

Abbreviation: MS = multiple sclerosis.

a Denominator is the number of patients with this information available.

b Assessed by orbital MRI, visual evoked potentials, and/or optical coherence tomography.

c Gadolinium-enhancing and nonenhancing lesions on initial MRI scan.

Most patients (67%) were referred because of a typical syndrome^2^ suggestive of MS: optic neuritis (n = 54), myelitis (n = 106), brainstem/cerebellar syndromes (n = 49), supratentorial syndromes (n = 11), or progressive myelopathy (n = 13). The remaining patients had atypical presentations including headache (n = 33), nonspecific sensory symptoms (n = 26), atypical optic neuropathies (n = 15), or myelopathies (n = 8), audio-vestibular symptoms (n = 8), and seizures/epilepsy (n = 3).

All patients underwent brain and spinal cord MRI, 298 (86%) had contrast-enhanced MRI, 265 (76%) had CSF examination, and 198 (57%) had optic nerve evaluation with orbital MRI (n = 66), VEPs (n = 149), and/or OCT (n = 50). Optic nerve evaluation was available in 63 (97%) patients with optic nerve symptoms and 135 (48%) patients without optic nerve symptoms. The findings of the baseline paraclinical evaluation are presented in Table 1.

Patients were followed up clinically and with MRI over a mean of 15.3 (4.0) months from initial clinical assessment. During follow-up, 98/314 (31%) patients had new T2 lesions on follow-up MRI.

### Exclusion of Other Disorders

In patients originally referred with suspicion of MS, 73 (21%) patients were identified with alternative diagnoses (Table 2), most commonly migraine or functional neurologic disorder in the context of non-specific/vascular white matter lesions on MRI. Two patients, 1 with seronegative neuromyelitis optica spectrum disorder and 1 with multifocal glioma had lesions in at least 2 regions typically affected in MS plus gadolinium-enhancing and nonenhancing lesions on MRI that could satisfy either the 2017 or 2024 McDonald criteria. However, the clinical and MRI findings included red-flag features that argued against a diagnosis of MS.

**Table 2:** Alternative Diagnoses in Patients Referred for Evaluation of Suspected Multiple Sclerosis

	Patients, n
Migraine	13
Functional neurologic disorder	13
Nonspecific sensory symptoms	7
Small vessel cerebrovascular disease	5
Spondylotic cervical myelopathy	4
Myelin oligodendrocyte glycoprotein antibody-associated disease	3
Neuromyelitis optica spectrum disorder	3
Cervical radiculopathy	2
Glioma	2
Ischemic stroke	2
Seronegative longitudinally extensive transverse myelitis	2
Peripheral neuropathy	2
Tumefactive demyelinating lesion	2
Chronic fatigue syndrome	1
Lumbar spinal stenosis	1
Myasthenia gravis	1
Neuroretinitis	1
Neurosarcoidosis	1
Recurrent isolated optic neuritis	1
Spinal dural arteriovenous fistula	1
Superficial siderosis	1
Trigeminal neuralgia	1
Viral meningoencephalitis	1
Visual snow	1

### Application of MS Diagnostic Criteria

**Figure 1:**
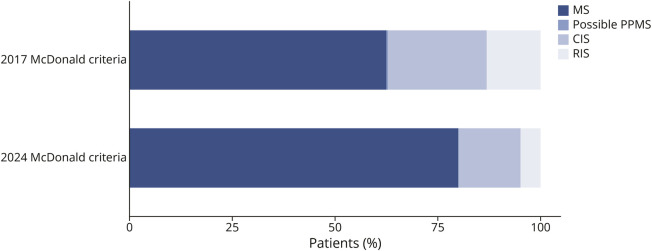
Diagnosis at the End of the Initial Diagnostic Workup in Patients With Suspected MS CIS = clinically isolated syndrome; MS = multiple sclerosis; PPMS = primary progressive MS; RIS = radiologically isolated syndrome.

After excluding patients with alternative diagnoses, the McDonald 2017 criteria were applied prospectively in the remaining 274 patients (Figure 1). After the initial diagnostic workup, 172 patients were diagnosed with MS (161 RRMS, 11 PPMS), and 102 patients had possible MS (66 CIS, 1 possible PPMS, 35 RIS).

The number of patients diagnosed with MS using the 2017 McDonald criteria at follow-up increased from 172 to 204 (192 RRMS, 12 PPMS); 28 patients with CIS had a second clinical attack and/or new T2 lesions at follow-up, 3 patients with RIS had a first clinical event, and 1 patient with possible PPMS had disability progression over >12 months.

The McDonald 2024 criteria were applied retrospectively using data from the initial diagnostic workup. The rate of MS diagnosis was 28% higher (220 vs 172 patients, *p* < 0.01) using the 2024 McDonald criteria compared with the 2017 criteria (Figure 1) because of application of the criteria in patients with RIS/nonspecific symptoms (n = 23), DIS in ≥4 typical regions (n = 15), and inclusion of optic nerve involvement in DIS (n = 10), including 9 patients with optic neuritis with abnormal orbital MRI and 1 patient with myelitis and abnormal VEPs. When the McDonald 2024 criteria were applied at last follow-up, the rate of MS diagnosis was 17% higher than with the 2017 criteria (237 vs 203 patients, *p* < 0.01).

The availability of paraclinical tests used to make a diagnosis of MS in different clinical scenarios is shown in eFigure 1. Application of the 2024 McDonald criteria at the end of initial diagnostic workup in patients with and without optic nerve evaluation, CSF examination and contrast-enhanced MRI scans is shown in eFigure 2. The increased rate of MS diagnosis was broadly similar in patients with and without each of the paraclinical tests.

**Figure 2:**
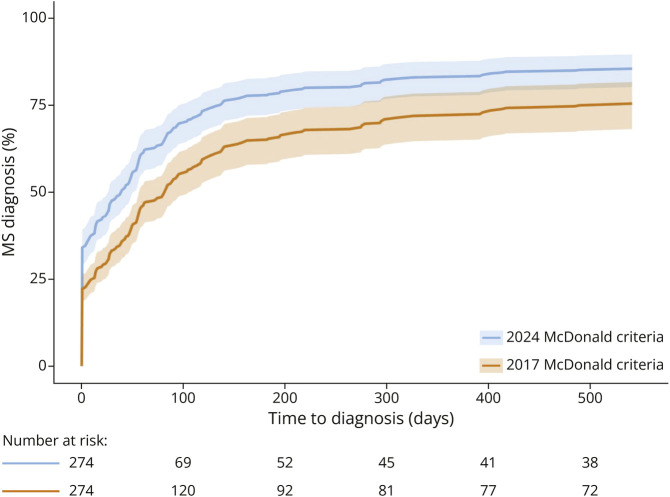
Time to Diagnosis of MS From First Clinical Evaluation MS = multiple sclerosis.

The higher rate of MS diagnosis using the 2024 McDonald criteria was confirmed in the shared frailty model (HR 1.78, 95% CI 1.45–2.18, *p* < 0.01). Time to MS diagnosis is shown in Figure 2; from the model the median time to MS diagnosis from first clinical evaluation was shorter using the 2024 vs 2017 McDonald criteria (40 vs 84 days).

### Patients Not Meeting the 2024 McDonald Criteria

There were 37 (13%) patients with clinical and/or MRI suspicion of MS, and no better explanation, who did not meet the 2024 McDonald criteria at the latest follow-up (mean 15.0 months). The availability of paraclinical in these patients is shown in eFigure 1.

Nine (3%) patients had DIS in ≥2/5 regions; optic nerve evaluation was missing in 1 patient with DIS in 2 regions. None of these patients had DIT on MRI at initial evaluation (8/9 patients had contrast-enhanced MRI) or at follow-up (all patients had ≥1 follow-up MRI), and OCBs were negative in all 7 patients tested. If MRI assessment for CVS had been available, some or all of these patients could potentially have been diagnosed with MS.

The remaining 28 (10%) patients had DIS in a single region; 23 patients had a first clinical attack and could potentially have been evaluated for CVS and/or PRLs to support a diagnosis of MS. However, most of these patients had a normal brain MRI (excluding any symptomatic lesion), and there were just 4 patients with brain lesions outside of the typical regions where these additional MRI tools could have been helpful, in combination with positive CSF and/or DIT on MRI.

### Performance of MS Diagnostic Criteria

The performance of the 2024 McDonald criteria when applied after the initial diagnostic workup is shown in Table 3. Using an MS diagnosis based on the 2017 McDonald criteria at follow-up as the outcome, the sensitivity (92.6%, 95% CI 88.1%–95.8%) and accuracy (83.6%, 95% CI 78.7%–87.8%) of the 2024 McDonald criteria was high, but the specificity was only moderate (57.8%, 95% CI 45.4%–69.4%).

**Table 3:** Diagnostic Performance of the McDonald 2024 Diagnostic Criteria

	Sensitivity, % (95% CI)	Specificity, % (95% CI)	Accuracy, % (95% CI)	PPV, % (95% CI)	NPV, % (95% CI)
McDonald 2024 criteria applied at the initial workup compared with the 2017 McDonald criteria at follow-up	92.6 (88.1–95.8)	57.8 (45.4–69.4)	83.6 (78.7–87.8)	86.2 (82.6–88.2)	73.2 (61.8–82.2)
McDonald 2024 criteria applied at the initial workup compared with new T2 lesions at follow-up	84.7 (76.0–91.1)	21.2 (16.3–29.0)	44.5 (38.6–50.6)	37.7 (35.1–40.5)	72.2 (60.2–81.7)

Abbreviations: NPV = negative predictive value; PPV = positive predictive value.

The findings were similar for new T2 lesions at follow-up with sensitivity (84.7%, 95% CI 76.0%–91.2%) but low specificity (22.2%, 95% CI 16.3%–29.0%). The timing of MRI follow-up was heterogeneous. We repeated the analysis in patients with follow-up MRI scans undertaken within 12 months of initial evaluation, and the findings were not materially different (data not shown).

When we repeated the performance testing in the whole cohort of patients referred with suspicion of MS without excluding patients with other disorders, the 2024 McDonald criteria continue to demonstrate high sensitivity and accuracy for a diagnosis of MS using the 2017 McDonald criteria (95.5%, 95% CI 91.6%–97.9%), or new T2 lesions (84.7%, 95% CI 76.0%–91.2%) at follow-up, but also higher specificity (77.9% [70.3%–84.2%] and 36.1 [29.7%–42.9%], respectively) (eTable 1).

### Special Patient Populations

#### Children <18 Years

In 27 children with suspected MS (5 [19%] patients <12 years), none had alternative diagnoses. A modestly higher number of patients were diagnosed with MS using the 2024 vs the 2017 criteria after initial diagnostic workup (15 vs 14, *p* = 1.0), and at follow-up (18 vs 17, *p* = 1.0). The 1 additional child diagnosed with MS had RIS with DIS and DIT/positive CSF. After initial diagnostic workup, the sensitivity of the 2024 McDonald criteria was high in children for an MS diagnosis using the 2017 McDonald criteria (82.4%, 95% CI 56.6%–96.2%) or new T2 lesions at follow-up (88.9%, 95% CI 51.8%–99.7%) (Table 4).

**Table 4:** Diagnostic Performance of the McDonald 2024 Diagnostic Criteria in Special Patient Populations

	Sensitivity, % (95% CI)	Specificity, % (95% CI)	Accuracy, % (95% CI)	PPV, % (95% CI)	NPV, % (95% CI)
McDonald 2024 criteria applied at the initial workup compared with the 2017 McDonald criteria at follow-up					
Children	82.4 (56.8–96.2)	90.0 (55.5–99.8)	85.2 (66.3–95.8)	93.3 (68.3–98.9)	75.0 (51.3–89.5)
Older adults	100 (90.5–100)	55.0 (31.5–76.9)	84.2 (72.1–92.5)	80.4 (71.7–87.0)	100 (71.5–100)
Vascular comorbidities	90.6 (75.0–98)	64.3 (35.1–87.2)	82.6 (68.6–92.2)	85.3 (74.0–92.2)	75.0 (48.8–90.4)
McDonald 2024 criteria applied at the initial workup compared with new T2 lesions at follow-up					
Children	88.9 (51.8–99.7)	61.1 (35.8–82.7)	70.4 (48.8–86.3)	53.3 (38.0–72.1)	91.7 (62.7–98.6)
Older adults	93.3 (68.1–99.8)	23.8 (12.1–39.5)	42.1 (29.1–55.9)	30.4 (26.1–35.2)	90.9 (58.3–98.6)
Vascular comorbidities	76.5 (50.1–93.2)	27.6 (12.7–47.2)	45.7 (30.9–61.0)	38.2 (30.5–46.7)	66.7 (41.4–85.0)

Abbreviations: NPV = negative predictive value; PPV = positive predictive value.

#### Older Adults ≥50 Years

In 88 older adults, 31 (35%) patients had alternative diagnoses and were excluded. In the older adults, the rate of MS diagnosis using the 2024 McDonald criteria was 39% higher after the initial diagnostic workup (46 vs 33 patients, *p* < 0.01), and 24% higher at follow-up (46 vs 37 patients, *p* < 0.01), compared with the 2017 criteria; 5 patients had RIS/nonspecific symptoms, 4 patients had DIS in ≥4 regions, and 1 patient only fulfilled DIS with optic nerve involvement. All older adults diagnosed with MS had abnormal spinal cord MRI, positive CSF, or both.

The performance of the McDonald 2024 criteria when applied after initial diagnostic workup in older adults in shown in Table 4. The sensitivity of the McDonald 2024 criteria for a diagnosis of MS using the 2017 criteria (100%, 95% CI 90.5%–100%) or new T2 lesions at follow-up (93.3%, 95% CI 68.1%–99.8%) was high, whereas the specificity was moderate-to-low (55.0%, 95% CI 31.5%–76.9% and 22.8%, 95% CI 12.5%–39.5%, respectively).

#### Patients With Vascular Comorbidities

In the whole cohort, 74 (21%) patients had vascular comorbidities and 28 patients had alternative diagnoses, and were excluded. The rate of MS diagnosis was higher in patients with vascular comorbidities using the 2024 McDonald criteria compared with the 2017 criteria after initial evaluation (34 vs 25 patients, *p* < 0.01), and at follow-up (38 vs 32 patients, *p* = 0.03), 4 patients had RIS/nonspecific symptoms and 2 patients had DIS in ≥4 regions.

When applied at the end of the initial diagnostic workup in patients with vascular comorbidities, the 2024 McDonald criteria displayed high sensitivity for a diagnosis of MS using the 2017 McDonald criteria (90.6%, 95% CI 75.0%–98.0%) or new T2 lesions (76.5%, 95% CI 50.1%–93.2%) at follow-up. Full results are presented in Table 4.

## Discussion

In this contemporary cohort of patients with evaluated for MS within routine clinical care, a higher number of patients could be diagnosed with MS after initial diagnostic workup using the 2024 McDonald criteria compared with the 2017 criteria, without further clinical and/or MRI follow-up. The overall number of patients diagnosed with MS was higher with the revised criteria, with fewer patients labeled as CIS or RIS. When applied at the end of the diagnostic workup, the new criteria displayed high sensitivity for an MS diagnosis using the 2017 criteria and/or new T2 lesions at follow-up, but the specificity was only moderate reflecting the higher rate of MS diagnosis including in nearly three-quarters of patients with RIS who cannot be diagnosed with MS using the 2017 criteria. Our findings suggest that when applied in routine clinical practice, use of the 2024 McDonald criteria will result in earlier and more frequent MS diagnosis.

The 2017 McDonald criteria enabled earlier MS diagnosis.^23^ However, only two-thirds of patients with CIS ultimately diagnosed with MS, satisfy the 2017 McDonald criteria at onset.^26^ Some patients lack evidence for DIS, others lack DIT or CSF analysis is either noncontributory, or not available, because of patient or health care system factors. Clinical and/or MRI follow-up is required to diagnose MS^23^ leading to delays in treatment. We confirm that the 2024 McDonald criteria revisions, particularly utilizing the inclusion of the optic nerve in the diagnostic criteria^7^ and allowing DIS alone,^9^ helps streamline MS diagnosis. These findings are in line with other recently published studies.^27,28^

People under evaluation for suspected MS do not always have a typical clinical syndrome—some patients have nonspecific symptoms^19,29^ and others have incidental MRI abnormalities suggestive of MS.^18^ Patients with RIS are at high-risk of a first clinical event (∼50% over 10 years),^18^ particularly younger patients and those with abnormal spinal MRI, positive OCBs/k-FLC, and elevated neurofilament.^18,30^ The 2024 McDonald criteria permit a diagnosis of MS in patients with RIS/nonspecific symptoms if there is DIS plus DIT, positive CSF or CVS, although careful application is essential and the risk of misdiagnosis might be higher in patients with atypical symptoms, or incidental MRI findings. We found that nearly three-quarters of patients with RIS satisfied the 2024 McDonald criteria using widely available diagnostic tools. In the remaining patients, none had DIT on MRI at the time of first evaluation or at follow-up, and CSF testing for OCBs was negative (available in 7 of the 9 patients). Had CVS assessment been available this might have further increased the number of patients with incidental MRI findings diagnosed with MS. These findings have significant implications for counseling and management of individual patients, particularly in those health care settings where patients with asymptomatic MS are not currently treated with disease-modifying therapies. The increased overall rate of MS diagnosis also raises concerns about overdiagnosis of MS.^31^ Epidemiologic studies have found largely stable MS incidence over the past 20 years,^32^ suggesting that the McDonald criteria promote earlier MS diagnosis rather than overdiagnosis, but continued surveillance is needed as diagnostic criteria evolve.

A novel feature of this study is the inclusion of both adults and children with suspected MS. The overall incidence of MS is lower in children and older adults,^32^ with special considerations for differential diagnosis.^20^ To address these challenges, the 2024 McDonald criteria provide additional recommendations for MS diagnosis in special patient populations, including routine MOG-IgG testing in younger children, and abnormal spinal MRI, positive CSF and/or CVS in older adults. We found that the new criteria have similar performance across the lifespan, and that the additional recommendations put forward for MS diagnosis in older adults do not adversely influence sensitivity, in-keeping with a recent study in patients with late-onset MS.^33^

Before applying MS diagnostic criteria it is essential to exclude alternative disorders.^6^ We found that approximately 1 in 5 patients referred with suspicion of MS were ultimately diagnosed with another condition, similar to a previous multicentre study.^34^ In total, 25 separate alternative diagnoses were made reflecting the broad differential diagnosis of suspected MS.^29^ The patients diagnosed with alternative disorders only infrequently had typical clinical presentations, but some had suggestive paraclinical findings, such as disseminated white matter lesions, spinal cord lesions, and/or positive CSF. Two patients had MRI changes that might satisfy the McDonald criteria for DIS, although the clinical and radiologic features were atypical. These observations highlight the enduring importance of the clinical assessment in patients with suspected MS,^29^ but also the potential promise of novel diagnostic biomarkers that can help differentiate MS from common mimics, avoiding misdiagnosis.^12,15^

A limitation of this study is that none of the patients were assessed for CVS, PRLs, or k-FLC, and only 60% of patients had optic nerve evaluation. These tests are not essential to diagnose MS if there is sufficient evidence of DIS and DIT with the other paraclinical tools,^6,22,35^ and their availability in a universal health care settings depends on local resources expertise. We therefore acknowledge a potential for ascertainment bias related to selective paraclinical testing, potentially underestimating the proportion of patients diagnosed with MS using the 2024 McDonald criteria. However, the cumulative benefit of these new paraclinical measures may be modest, for example, among nonoptic neuritis patients missing optic nerve evaluation, >85% already had sufficient evidence for DIS to make a diagnosis of MS. Therefore, optic nerve evaluation would have been helpful in the remaining 13 patients. Assuming ∼20% sensitivity for detection of asymptomatic optic nerve lesions using VEPs/OCT, another 2 or 3 patients might have fulfilled DIS criteria.^7,36^ Similarly, a minority of patients who did not already meet the 2024 McDonald criteria might have benefited from CVS assessment; 9 patients had DIS without DIT on MRI/negative CSF and only 4 patients had involvement of a single topography and brain white matter lesions that could have been assessed for CVS (or PRLs). Although the overall number of patients diagnosed with MS might have been modestly higher with CVS assessment, the effect could be much greater on time to diagnosis of MS since iron-sensitive sequences can be obtained at the same time as conventional MRI used to establish DIS,^13^ avoiding the need for follow-up MRI scans and/or lumbar puncture.^11^

Some further limitations of this study should also be noted. First, all of the patients included were evaluated before the publication of the 2024 McDonald criteria. Now that the criteria are being routinely applied in clinical settings, practice patterns may change as new diagnostic biomarkers are implemented.^35^ The retrospective nature of this study may underestimate the effect of the 2024 McDonald criteria on practice going forward once all the new paraclinical tools in the new diagnostic framework are used prospectively. Second, availability of diagnostic testing for MS and how the McDonald criteria are applied varies based on geography, health care system and the experience of individual neurologists.^37,38^ Although this study included patients seen within routine clinical care, rather than as part of a structured research cohort, the patients were seen at 2 specialist centers and findings might not be generalizable to all practice settings. Finally, the lack of a “gold-standard” when evaluating the performance of the McDonald criteria needs to be acknowledged, although this limitation is common to most diagnostic validation studies in MS. Historically, a second attack (i.e., clinically-definite MS) has been used as the gold-standard^39^ but this is not feasible in contemporary studies because patients are now routinely diagnosed (and treated) before a second attack.^40^ Furthermore, a relapse is not useful for evaluating PPMS diagnostic criteria.^21^ For these reasons we did not feel it was appropriate to apply the Poser criteria as a gold-standard. Instead, we used an MS diagnosis using the 2017 McDonald criteria and/or new T2 lesions at follow-up as references-standards, in line with other recent studies.^7^ The sensitivity of the 2024 criteria for these outcomes was high but the “specificity” was moderate. This needs to be interpreted with caution because specificity here does not refer to absence of a disease, since other disorders have already been excluded. Instead, the reduced specificity reflects the increased rate of MS diagnosis using the 2024 criteria with patients with RIS and CIS not meeting the 2017 criteria considered “false-positives.” The duration of follow-up in our study was also short (∼15 months). Although evaluating a contemporary patient cohort is a potential strength of this study, with longer follow-up some patients might satisfy both the 2017 and 2024 criteria. Although not intended to differentiate MS from other disorders, it is reassuring that the specificity of the 2024 criteria was nearly 80% when tested in all patients referred with suspicion of MS, including patients ultimately diagnosed with other disorders.

In conclusion, we found that the 2024 McDonald criteria allow for earlier and more frequent MS diagnosis in patients under evaluation for suspected MS. The new criteria perform well in children and older adults. Future studies should evaluate the application and performance of the 2024 McDonald criteria in prospective cohorts, ideally incorporating all of the novel diagnostic biomarkers included in the updated criteria.

## Glossary

CIS: clinically isolated syndrome
CVS: central vein sign
DIS: dissemination in space
DIT: dissemination in time
HR: hazard ratio
k-FLC: kappa free light chain
MOG-IgG: myelin oligodendrocyte glycoprotein immunoglobulin G
MS: multiple sclerosis
NPV: negative predictive value
OCB: oligoclonal band
OCT: optical coherence tomography
PPMS: primary progressive MS
PPV: positive predictive value
PRL: paramagnetic rim lesion
RIS: radiologically isolated syndrome
RRMS: relapsing-remitting MS
VEP: visual evoked potential
